# A method for the three-dimensional reconstruction of Neurobiotin™-filled neurons and the location of their synaptic inputs

**DOI:** 10.3389/fncir.2013.00153

**Published:** 2013-10-01

**Authors:** Matthew J. Fogarty, Luke A. Hammond, Refik Kanjhan, Mark C. Bellingham, Peter G. Noakes

**Affiliations:** ^1^School of Biomedical Sciences, The University of QueenslandBrisbane, QLD, Australia; ^2^Queensland Brain Institute, University of QueenslandBrisbane, QLD, Australia

**Keywords:** motor neuron, dendrite, 3D-reconstruction, synapse, brainstem

## Abstract

Here, we describe a robust method for mapping the number and type of neuro-chemically distinct synaptic inputs that a single reconstructed neuron receives. We have used individual hypoglossal motor neurons filled with Neurobiotin by semi-loose seal electroporation in thick brainstem slices. These filled motor neurons were then processed for excitatory and inhibitory synaptic inputs, using immunohistochemical-labeling procedures. For excitatory synapses, we used anti-VGLUT2 to locate glutamatergic pre-synaptic terminals and anti-PSD-95 to locate post-synaptic specializations on and within the surface of these filled motor neurons. For inhibitory synapses, we used anti-VGAT to locate GABAergic pre-synaptic terminals and anti-GABA-A receptor subunit α1 to locate the post-synaptic domain. The Neurobiotin-filled and immuno-labeled motor neuron was then processed for optical sectioning using confocal microscopy. The morphology of the motor neuron including its dendritic tree and the distribution of excitatory and inhibitory synapses were then determined by three-dimensional reconstruction using IMARIS software (Bitplane). Using surface rendering, fluorescence thresholding, and masking of unwanted immuno-labeling, tools found in IMARIS, we were able to obtain an accurate 3D structure of an individual neuron including the number and location of its glutamatergic and GABAergic synaptic inputs. The power of this method allows for a rapid morphological confirmation of the post-synaptic responses recorded by patch-clamp prior to Neurobiotin filling. Finally, we show that this method can be adapted to super-resolution microscopy techniques, which will enhance its applicability to the study of neural circuits at the level of synapses.

## Introduction

The number, distribution, and neuro-chemical type of synaptic inputs that a postsynaptic neuron receives strongly influences its functional output, which is then passed onto its target cell. Localizing and quantifying the synaptic inputs received by an individual neuron is a vital step toward understanding neural circuit function. Although reasonable morphological approaches do exist to quantify the number of synapses present on a neuron, including their neuro-chemical type (e.g., glutamatergic excitatory synapses; (Segal, 2005; Tada and Sheng, 2006; Harms and Dunaevsky, 2007), there remains a need to more easily and rapidly quantify the number and type of synapses on an identified neuron that has also been electrophysiologically analyzed in its *in vivo* setting.

To date, the location and distribution of synapses on neurons that have been morphologically analyzed have been determined by processes such as electron microscopy (EM; Bae et al., 1999; Megias et al., 2001; Shigenaga et al., 2005; Arthur et al., 2007; Chen et al., 2008b), or by light microscopy of cultured neurons. However, these techniques are limited by the lack of three-dimensional morphology of the entire post-synaptic neuron, replete with all of the surrounding cellular inputs in its physiological setting [e.g., cultured neurons; (Cullen et al., 2010; Ivenshitz and Segal, 2010; Schatzle et al., 2012)], as well as the limited and labor-intensive nature of being able to identify the synaptic type, including the molecular make up of its postsynaptic specialization [i.e., neurotransmitter type or post-synaptic adaptor proteins, as in EM analyses; (Chen et al., 2008b; Dani et al., 2010)]. Previous work in the *Manduca* (Meseke et al., 2009) and *Drosophila* (Tripodi et al., 2008) nervous systems has been able to produce highly accurate three-dimensional reconstructions of motor neurons and pre-synaptic components closely opposed to the neuron. Methodologies and recommendations for accurate and automatic reconstruction of dendritic trees have been outlined for Purkinje cells, cultured astrocytes, locust sensory neurons and *Manduca* motor neurons (Evers et al., 2005). None of the existing methods were used to correlate electrophysiological analysis of individual neurons with their individual high resolution morphology (i.e., cell size, cell surface contours including dendrites and post-synaptic processes) as well as marking the number and distribution of synaptic inputs onto the functionally assessed neuron for comparison to electrophysiological recording of excitatory and inhibitory post-synaptic currents.

Here we present a semi-automated method for mapping the number and type of synaptic inputs that a single reconstructed neuron receives in thick (300 μm) *ex vivo* brainstem slices. Our technique preserves the cell's *in vivo* size, shape, dendritic arbor, surrounding synaptic inputs, and the local macro-architecture of the brainstem. Importantly, it allows for subsequent immuno-labeling and semi-automated computer image analysis to rapidly map and characterize the synaptic inputs that the filled neuron receives. Here, in mice, an increasingly important species not only for basic neuroscience research, but also a preclinical model for human disease, we present a method to determine the functional and morphological excitatory and inhibitory synaptic inputs received by hypoglossal motor neurons, during the developmental stage at postnatal day 0 (P0) when these motor neurons are forming synaptic connections with their target muscle, the tongue (Banks et al., 2005; Fogarty et al., 2013).

In particular, we provide a validation of our method for quantifying the number and distribution of glutamatergic and GABAergic synapses made on hypoglossal motor neurons from C57-Bl6 mice at birth. Hypoglossal motor neurons were filled with Neurobiotin™ as part of patch clamp recordings and visualized with Cy3-Streptavidin that binds to Neurobiotin. The brainstem sections with high-quality motor neuron fills were then double immuno-labeled for glutamatergic or GABAergic synaptic terminal endings and for markers of glutamatergic or GABAergic postsynaptic specializations, using extensively validated and commercially available pre- and post-synaptic marker antibodies. For glutamatergic pre-synaptic endings, we have used anti-vesicular glutamate transporter type 2 (VGLUT2), a member of the family of vesicular transporters of glutamate principally associated with neurotransmission at excitatory synapses (Wallen-Mackenzie et al., 2010). For excitatory post-synaptic specializations, we have used anti-post-synaptic density 95 (anti-PSD-95), which labels PDS-95, a scaffold protein that interacts with post-synaptic glutamate receptors and dendritic spine cytoskeletons in the post-synaptic membrane of neurons (Sheng and Pak, 2000; Sheng and Sala, 2001). For the GABAergic pre-synaptic terminals, we have used antibody against VGAT (vesicular inhibitory amino acid transporter) a GABA/glycine co-transporter, prevalent in both GABAergic and glycinergic inhibitory neurons, localizing to the synaptic vesicles within the terminal ends of these neurons (McIntire et al., 1997; Chaudhry et al., 1998; Dumoulin et al., 1999). For inhibitory post-synaptic specializations, we have used antibody against GABA-A receptor subunit α1, a post-synaptic receptor component of inhibitory GABA-A synapses (Vicini et al., 2001). These primary antibodies have been applied to the mouse central nervous system and validated extensively, VGLUT2 (Graziano et al., 2008; Jakovcevski et al., 2009), PSD-95 (Gazula et al., 2010; Soiza-Reilly and Commons, 2011; Spangler et al., 2011), VGAT (Dudanova et al., 2007; Panzanelli et al., 2007; Fortune and Lurie, 2009; Jakovcevski et al., 2009) and GABA-A receptor subunit α1 (Panzanelli et al., 2007; Belichenko et al., 2009; Patrizi et al., 2012). This subsequently allowed us to use commercially available software Imaris (BitPlane, South Windsor, CT, USA) to reconstruct the filled neuron and to determine the distribution and number of glutamatergic or GABAergic synapses contacting it.

This method outlines the practical aspects of using commercial imaging software such as Imaris, to provide a rapid and robust glimpse of how the synaptic inputs received by a single post-synaptic neuron can be quantified morphologically, following electrophysiological recordings of synaptic currents from the same neuron. We also show how our method can take advantage of increasingly powerful and prevalent super-resolution imaging options.

## Materials and methods

### Ethics statement

All experimental procedures were approved by the University of Queensland Animal Ethics Committee (Permit Numbers: 227-09, 924-08, and 152-12), and complied with the policies and regulations regarding animal experimentation and other ethical matters (Drummond, 2009). They were conducted in accordance with the Queensland Government Animal Research Act 2001, associated Animal Care and Protection Regulations (2002 and 2008), the Australian Code of Practice for the Care and Use of Animals for Scientific Purposes, 7th Edition (National Health and Medical Research Council, 2004).

### Slice preparation and neurobiotin cell filling

Wild type C57BL/6 mice were used in this study, at postnatal day 0 (birth/P0). Cell filling was done using a semi-loose seal electroporation method first developed by us for the retina (Kanjhan and Vaney, 2008) and later adapted to our studies on brainstem hypoglossal motor neurons (Kanjhan and Bellingham, 2013). Briefly, P0 pups were collected immediately after birth. Pups were anesthetized by hypothermia for ~3 min, following by the removal of scalp and underlying bones, and decerebration rostral to the pons. They were then placed in ice-cold modified (high-Mg^2+^) Ringer solution [see (Kanjhan and Bellingham, 2013) for details] continuously bubbled with 95% O2/5% CO_2_ to maintain pH at 7.4. The brainstem and the cervical spinal cord were rapidly dissected from the surrounding tissues in ice-cold Ringer slurry. Transverse brainstem sections (300 μm; 4 sections per animal) were cut on a vibratome (DSK Microslicer, Ted Pella Inc, Redding, CA, USA), in an ice-cold bath of the modified Ringer solution. Slices were incubated for 45 min in modified (high-Mg^2+^) Ringer solution at 34°C. The sections were then transferred to normal Ringer solution (Kanjhan and Bellingham, 2013) continuously bubbled with 95% O2/5% CO_2_ and maintained at room temperature (20–22°C) for 30 min prior to recording and Neurobiotin filling. Electrodes were pulled from borosilicate glass capillaries (Modulohm, Vitrex Medical, Denmark) and were filled with pipette solution containing 2% Neurobiotin™ (Vector Labs, Burlingame, CA, USA) in a potassium or cesium methane sulphonate intracellular solution [see (Kanjhan and Vaney, 2008) for details]. Under visual guidance (Zeiss Axoskop II), patch electrodes were maneuvered against the soma using a manipulator (MPC-200, Sutter Instrument Company, USA), and a low negative pressure was applied until a stable membrane seal of >50 MΩ was obtained. Square wave voltage pulses (+20 to 50 mV; equivalent of ~300–500 pA) of 500 ms duration at 1 Hz maintained for 5–6 min were used to electroporate the membrane and fill the motor neuron with Neurobiotin. The voltage pulses often resulted in establishment of whole cell recording conditions by membrane breakdown in cells with a giga-Ohm seal, as seen by a large drop in the membrane resistance, and by appearance of spontaneous synaptic currents and action currents at a holding potential of −60 mV. Upon completion of whole cell recording, square wave voltage pulses as above were re-applied to ensure that the cells were filled with Neurobiotin. Up to 2 cells on each side of the brainstem section were filled with Neurobiotin.

### Immunocytochemistry

After filling, the sections were left to rest in the recording bath for a minimum of 10 min to allow diffusion of Neurobiotin. Sections were then fixed in 4% paraformaldehyde in 0.1 M phosphate buffer (pH 7.4) for 20–30 min at room temperature, and subsequently washed 3–4 times for 30 min in 0.1 M phosphate-buffered saline pH7.4 (PBS) at 4°C. Sections were incubated to block non-specific background labeling for 2 h in PBS containing 2% bovine serum albumin (BSA) and 0.05% Triton-X 100 at 4°C. Sections were then incubated for 2–4 h at 4°C in Cy3-Streptavidin (Sigma; 1:500 in blocking solution) to visualize Neurobiotin. The labeling quality of cellular morphology was quickly checked using a basic fluorescence microscope with sections mounted with PBS without a coverslip; only neurons with exceptionally good fill were included for further study. To detect glutamatergic pre-synaptic components, slices were incubated with rabbit anti-VGLUT2 (Synaptic Systems, Goettingen, Germany) and for the post-synaptic domain with mouse anti-PSD-95 (Synaptic Systems) at 4°C for 48 h. Both primary antibodies were diluted at 1:250 in 0.1 M PBS/2% BSA/0.02% Triton-X 100. Slices were washed three times in 0.1 M PBS prior to incubation with a secondary antibody cocktail consisting of 1:500 anti-rabbit alexa-488 (Invitrogen, Mulgrave, Vic, Australia) plus 1:500 anti-mouse Cy5 (Millipore, Billerica, MA, USA) in 0.1 M PBS/2%BSA at 4°C for 24 h. To detect GABAergic synapse components in other tissue slices, a mouse anti-VGAT (Synaptic Systems) and rabbit anti-GABA-A α1 subunit (Synaptic Systems) primary antibody cocktail was used. Both primary antibodies were diluted at 1:250 in 0.1 M PBS/2% BSA/0.02% Triton-X 100, washed in PBS and incubated with secondary antibodies (anti-rabbit alexa-488, Invitrogen; plus anti-mouse Cy5, Millipore), at a dilution of 1:500 in 0.1 M PBS/2%BSA at 4°C for 24 h.

We also conducted two double immuno-labeling control experiments on Cy3-Neurobiotin filled neurons to validate that our method was robust in being able to identify the type of synapses (excitatory or inhibitory) being formed on motor neurons. In these experiments, slices containing Cy3-Neurobiotin filled motor neurons were incubated in either a cocktail of rabbit anti-VGLUT2 plus mouse anti-VGAT, or incubated in a mixture of rabbit anti-GABA-A α1 subunit plus mouse anti-PSD-95 for 48 h at 4°C. As described above, these primary antibodies were diluted at 1:250 in 0.1 M PBS/2% BSA/0.02% Triton-X 100 and washed in PBS. Slices were then incubated with secondary antibodies (anti-rabbit alexa-488, Invitrogen; plus anti-mouse Cy5, Millipore), at a dilution of 1:500 in 0.1 M PBS/2%BSA at 4°C for 24 h. Control experiments included incubating preparations in secondary antibody only, where no punctate staining was observed (not shown).

Subsequently, all slices were washed in 0.1 M PBS 3 times for 15 min, mounted on slides (Menzel-Glaser, Braunschweig, Germany) in a standard glycerol-based *p*-phenylenediamine mounting medium (10 mg *p*-phenylenediamine in solution of 9 ml glycerol and 1 ml of 0.1 M phosphate buffer) and cover-slipped (0.17 mm thick, Menzel-Glaser).

### Confocal imaging and image analysis

Sections were imaged with a Zeiss LSM 510 Meta confocal microscope (Carl Zeiss, Oberkochen, Germany) equipped with an argon laser having a 488 nm emission, one helium neon laser with 543 nm emission and a second helium neon laser with a 633 nm emission. Scanning was sequential with a 1.6 μs dwell time. Images were sampled at a resolution of 1024 by 1024 pixels, using a 63x (NA 1.4) objective, a 2.5 times software zoom and a *z*-step size of 0.3 μm. Based on the fluorescent probes used, the maximum optical resolution was 150 nm in the *xy*-plane and 560 nm in the *z*-plane. Chromatic aberration was assessed using microscopic beads and no distortion was found. Images were saved in the “lsm” format and quantification was performed on a Dell T5600 Dual Socket Intel Xeon E5607 2.26 GHz PC workstation with a 6 GB NVIDIA graphics card and 128 GB DDR3 ECC RDIMM 800 MHz RAM using Imaris 7.6.1 (Bitplane, South Windsor, CT, USA) and MatLab 8.0 (Mathworks, Natick, MA, USA).

### Structured illumination super-resolution microscopy

Sections were imaged using a Zeiss ELYRA PS.1 instrument (Carl Zeiss, Jena, Germany) using an AndoriXon 885 EMCCD camera (Andor, Belfast, Northern Ireland) and a Zeiss 63x/1.46 NA Plan Apochromatic objective. Each fluorescent channel was acquired using five pattern rotations with 5 translational shifts. The final image for each channel was then reconstructed using Zen 2011 (Carl Zeiss, Jena, Germany) and analyzed with Imaris as above.

### Electrophysiology analysis

Electrophysiological recording of spontaneous synaptic currents was carried out after establishing whole cell recording conditions. Axograph 3.5 (Axon Instruments, Foster City, CA) and a Macintosh G3 computer was used to record epochs of data at different holding voltages, via a Digidata 1322 digitizer (sampling frequency 5–10 kHz, low pass filter 2 kHz). Synaptic currents were detected and analyzed offline, using the optimally scaled sliding template algorithm (Clements and Bekkers, 1997) in Axograph X (www.axograph.com). Measured parameters of detected synaptic currents were imported into Microsoft Excel (Microsoft, Redmond, WA) and Prism 5 (Graphpad, San Diego, CA) for further statistical analysis. Fast rising (0.5–1 ms) and decaying (<10 ms) inward currents were frequent at -60 mV, while slower rising (1–2.5 ms) and decaying (>10 ms) outward currents present at 0 mV were less frequent. At intermediate voltages, a mixture of inward and outward currents was present. Due to the respective calculated reversal potentials for EPSCs (−4 mV) and IPSCs (−69 mV), inward currents at −60 mV were glutamatergic, while outward currents were GABA-A/glycinergic at 0 mV.

### Statistical methods

Mean and standard error of the mean (SEM) or 95% confidence intervals were calculated for each data set. Where indicated, unpaired two tailed *t*-tests were conducted for all analysis involving the comparison of group means, using Prism 5, while the Kolmogorov–Smirnov test was used to compare cumulative frequency distributions (Axograph X). Statistical significance was accepted at *P* < 0.05.

## Results

Our aim was to reconstruct the hypoglossal motor neuron that had been Cy3-Neurobiotin filled and subsequently immuno-labeled to identify its glutamatergic or GABAergic synapses. To achieve this, we first optically sectioned the entire filled hypoglossal motor neuron, which was largely confined to the top 50 μm of a single brainstem section, using either confocal or super resolution microscopes, at 0.3 μm z–step intervals. These microscopes were equipped with lasers that excited Cy3, Alexa488, and Cy5 fluorophores. This allowed us to illuminate Cy3-fluoroscent hypoglossal motor neurons, the distribution of Alexa488-fluoroscent labeled VGLUT2 synaptic endings, and Cy5-fluoroscent labeled PSD-95 membrane domains, in these sections. In the case of slices assayed for GABAergic synapses, the distribution of Alexa488-fluoroscent labeled GABA-A α1 receptor subunit post-synaptic regions, and Cy5-fluoroscent labeled VGAT terminals was imaged. The Cy3, Alexa488, and Cy5 fluorescent information within these optical sections were then captured and digitized. We then employed Imaris imaging software to reconstruct the hypoglossal motor neuron, including the distribution of its glutamatergic and GABAergic synapses. We chose Imaris, as the software is integrated with distance-detection Matlab algorithms, automatic detection of objects in 3-dimensional space based on both intensity and size, and the ability to project these onto complex structures such as dendrites. Other software programs such as FIJI (open source, available at http://fiji.sc/Fiji) and MetaMorph (Molecular Devices, Sunnyvale, CA, USA) may provide similar results. What follows is a step-by-step description of how this was done using Imaris.

### The creation of a neuronal surface

**Figure 1:**
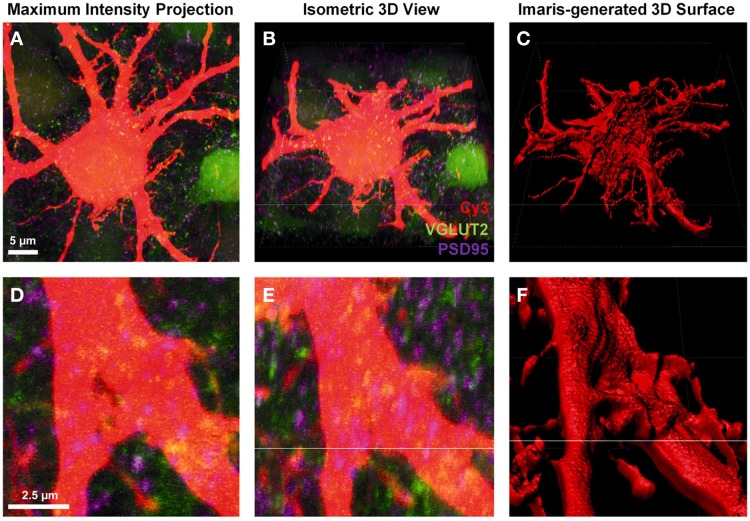
**Creating a neuronal surface. (A,D)** show low and high magnification the maximum intensity projection of a Neurobiotin-filled hypoglossal motor neuron (Cy3) and excitatory synaptic components VGLUT2 (green) and PSD-95 (purple). **(B,E)** show low and high magnification 3D isometric view of the same neuron in Imaris. **(C,F)** show low and high magnification of the neuronal surface created in Imaris using the “*create surface*” tool. Scale bars: (**A,B,C)** 5 μm and **(D,E,F)** 2.5 μm.

To create a detailed surface rendering of the Neurobiotin-filled motor neuron we used the *z*-stack fluorescent images generated from confocal microscopy. In this case, a Cy3-Neurobiotin filled motor neuron (red), glutamatergic excitatory pre-synaptic terminals labeled with anti-VGLUT2 localized with Alexa488 secondary antibody (green), and glutamatergic post-synaptic densities labeled with anti-PSD-95 localized with Cy5 secondary antibodies (depicted as purple in all figures). The *z*-stack images were displayed as a conventional 2-dimensional maximum intensity projection image (Figures 1A,D). Loading this image into the Imaris software projects the display as three-color channels of confocal *z*-stack fluorescence in a 3-dimensional isometric view (Figures 1B,E). From here, we were only interested in the Cy3-Neurobiotin neuronal fill, to create a 3D surface defining the cellular domain using Imaris software (Figures 1C,F). This neuronal surface was used to demarcate the labeled neuron's post-synaptic domain from the pre-synaptic domain input from other neurons.

The “*create surface*” tool was used to make a solid surface best matching the neuron anatomy. To do this, we used Cy3 labeling of a Neurobiotin-filled motor neuron and dendrites as the source channel (Figures 1A,B,D,E). The “*smoothing*” tool was disabled, as this introduces an artificial uniformity to the cell surface. As our sections were thick and we were trying to detect fluorophores up to 50 μm deep from the surface of the slice, the background subtraction option was enabled. The minimum diameter setting was determined by selecting “*slice view*” and using the line tool to measure the diameter of the smallest dendrite found in the confocal stack.

Next, using the interactive software histogram of Cy3 voxels (volumetric pixels), a threshold was selected so as to include as much of the neuron as possible while excluding any background. When choosing a correct threshold, which is low enough to accurately demarcate the neuron of interest, small artifacts appearing as small blebs consistent with background fluorescence, which occasionally appeared outside the labeled cell soma and dendrites. These were filtered out based on size in the subsequent step by setting a minimum object size in the interactive size threshold histogram, the minimum size being related to the volume of the labeled cell soma or dendrite.

### Filtering pre-synaptic and post-synaptic channels related only to the filled neuron

**Figure 2:**
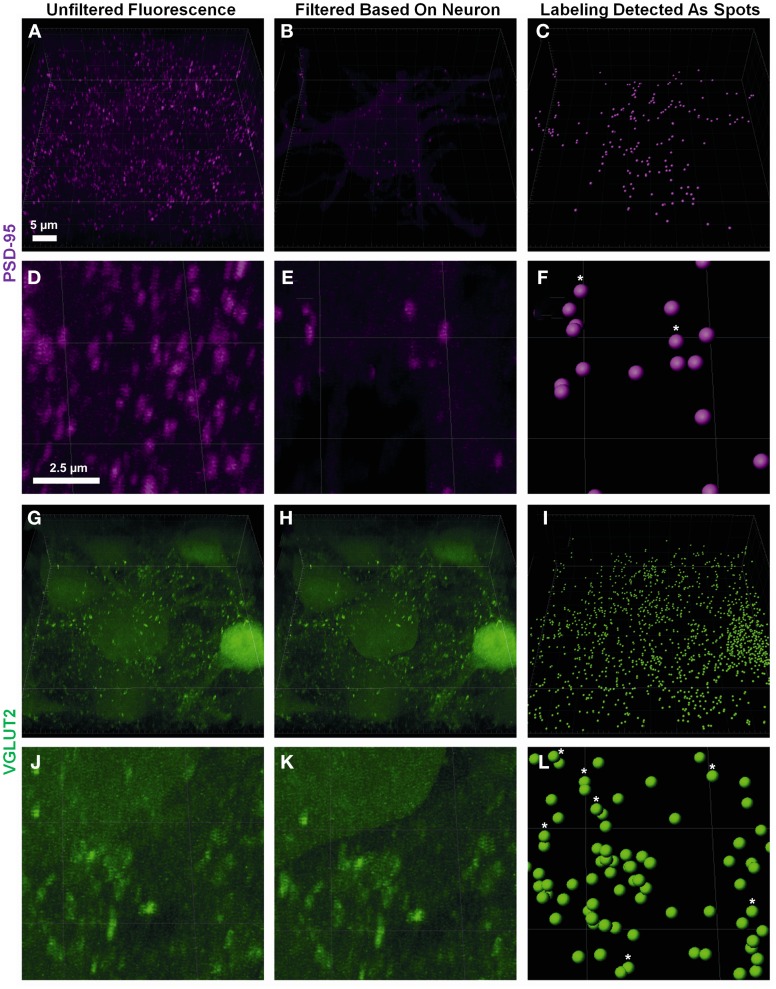
**Defining pre- and post-synaptic spots. (A,D)** show low and high power magnification of post-synaptic PSD-95 (purple) fluorescence in the entire confocal *z*-stack from Figure 1. **(B,E)** show the post-synaptic PSD-95 (purple) fluorescence filtered to inside the neuronal surface using the “*mask all*” tool in Imaris. **(G,J)** show low and high power magnification of pre-synaptic VGLUT2 (green) fluorescence in the entire confocal *z*-stack from Figure 1. **(H,K)** show the pre-synaptic VGLUT2 (green) fluorescence filtered to outside the neuron surface using the “*mask all*” tool in Imaris. **(C,F,I,L)** show low and high-powered magnifications of post-synaptic PSD-95 (purple) and pre-synaptic VGLUT2 (green) identified using the “*create spots*” algorithm in Imaris. Scale bars: **(A,B,C,G,H,I)** 5 μm and (**D,E,F,J,K,L)** 2.5 μm.

Pre-synaptic and post-synaptic color channels were then filtered based on the neuronal surface, so that only the fluorescence relevant to pre-and post-synaptic elements of the filled neuron remained. This required removal of all pre-synaptic fluorescence signals inside the filled cell, and all the post-synaptic fluorescence outside the filled cell. In the confocal *z*-stack, PSD-95 post-synaptic marker labeling was distributed throughout the whole field of view (Figures 2A,D). As we were only interested in the PSD-95 staining associated with the surface of the filled neuron, we filtered out the *z*-stack (and the data set) of all PSD-95 staining outside the boundary of the cell surface, conserving only the portion of the signal which was continuous with the neuron's cell surface (Figures 2B,E). For the pre-synaptic marker VGLUT2, we also observed labeling throughout our entire *z-stack* confocal field (Figures 2G,J). In this case, only VGLUT2 labeling outside our filled neuron was of interest for analysis. We therefore filtered out of the *z*-stack (and data set), all VGLUT2 labeling continuous with the cell surface and staining within the filled neuron (i.e., its membrane and cytoplasm; Figures 2H,K).

Fluorescent channels were filtered by selecting the newly created neuron surface layer and using the edit tab; we selected “*mask all*.” For pre-synaptic markers we enabled “*duplicate channel before applying mask*” to preserve the original data, selected “*constant inside/outside*” and checked “*Set voxels INSIDE surface to 0.0*.” This removed all pre-synaptic labels continuous with the cell membrane and within its cytoplasm. For post-synaptic markers, we repeated these steps, but set the mask option to “*Set voxels OUTSIDE surface to 0.0*.” This removed all post-synaptic fluorescent signals extraneous to the cell surface and cytoplasm (Figures 2B,E,H,K).

### Creating discrete pre- and post-synaptic quantitative “spots” from filtered channels

Spot analysis allowed for proper quantification of the appropriately filtered pre-synaptic and post-synaptic components of the labeled cell. There were two important considerations for the quantification of synaptic components. The first was having well-defined puncta that were of a minimum diameter (usually measuring between 0.25 and 0.8 μm (Chen et al., 2008a; Kim and Sheng, 2009; Dumitriu et al., 2012). If the threshold for punctate diameter was too low, the possibility of background or scattered fluorescence being detected as a quantifiable synaptic component increased. The second important thresholding concern was the relative intensity of the punctate staining compared to the background. As we were capturing thick confocal *z*-series using oil objectives, the background fluorescence as we imaged deeper into the slice increased substantially (Smith, 2011). Our analysis utilized a background subtraction function within the Imaris software, which measured the mean intensity of the fluorescence of all low-contrast regions at different *z*-depths, and then uniformly subtracted this intensity from the fluorescence level used by the Imaris algorithm to detect punctate staining “spots.” Thus spots that were unable to be discerned by the naked eye due to higher background fluorescence deeper into the sample, were quantified by the Imaris software as a “spot” (as seen in Figure 2, stars in panels **C,F,I,L**). It is also important to have a minimum puncta diameter that is larger than the *z* step-size to ensure puncta are present in a minimum of two confocal optical slices. This helps to ensure that these puncta exist in 3D space and are not artifacts of the background subtraction algorithm.

Using the Imaris software we created a new “*spots*” layer, selected the pre-synaptic label channel and enabled background subtraction. Next, we determined the smallest diameter of the representative labeling: as in the neuron surface creation (section The Creation of a Neuronal Surface), the spot diameters were measured by switching to “*slice view*” and measuring with the line tool. In our study we chose a spot diameter of 0.3 μm, based on past studies (Nahmani and Erisir, 2005). Next, sensitivity for detecting spots was adjusted using the automatically-generated interactive histogram based on voxel size, with minima determined by previously nominated spot diameters. We selected an area on the histogram to accurately detect as many spots as possible without creating artifacts. Moving the histogram to the left decreased the minimum size threshold used to detect spots. In the case of PSD-95, this was often done to account for the lower diameter puncta that make up approximately 50% of all PSD-95 aggregations (Dumitriu et al., 2012). Generally the point of inflection (i.e., where the histogram changed from a slope of high magnitude to low magnitude (plateaus) on the graph yielded the best results; in the example shown here, we chose 13.5 arbitrary units (Figures 2I,L). We repeated this process for the post-synaptic label using a diameter setting of 0.6 μm, lying within the theoretical distribution of previous studies (Chen et al., 2008a; Waataja et al., 2008; Kim and Sheng, 2009; Dumitriu et al., 2012) and 14.9 arbitrary units on the aforementioned voxel size threshold histogram (Figures 2C,F).

### Analysis of pre- and post-synaptic localizations

**Figure 3:**
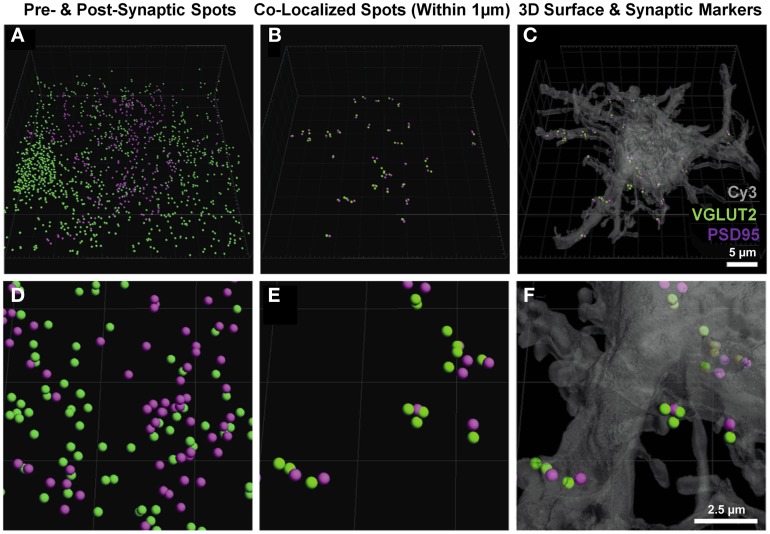
**Pre- and post-synaptic localizations to the motor neuron. (A,D**) show the pre-synaptic VGLUT2 (green) and post-synaptic PSD-95 (purple) Imaris-generated “spots” from the neuronal surface masked fluorescence in Figure 2. **(B,E)** show these spots co-localized to within 1 μm using the “co-localize spots” algorithm in Imaris. As all of the post-synaptic elements (PSD-95, purple) are already filtered to be on the neuron, we get a quantification of pre- (VGLUT2, green) and post-synaptic components (PSD-95, purple) with regard to the Neurobiotin-filled hypoglossal motor neuron (shown in transparent gray-Cy3) in **(C)** and **(F)**. Scale bars: **(A,B,C)**, 5 μm and **(D,E,F)** 2.5 μm.

Once the neuron-specific pre- and post-synaptic labeled spots were detected, two analytical options became available; (i) the total number of pre-synaptic spots adjacent (e.g., within 1 μm) to the plasma membrane of the filled neuron, and (ii) analysis of pre- and post-synaptic spots opposed to each other, providing for an estimate of synapse number. The second analysis involved the co-localization of pre-synaptic components (from multiple unknown sources) to the post-synaptic domains of the filled neuron. Imaris software was used to determine which of the pre- and post-synaptic markers (shown without co-localization in Figures 3A,D) were within a threshold distance of each other; in this case, we determined which VGLUT2 pre-synaptic endings were within 1 μm of PSD-95 post-synaptic specializations of the filled neuron (Figures 3B,E). These co-localized spots were then superimposed onto the filled neuronal surface, to provide their location and distribution (Figures 3C,F).

**Figure 4:**
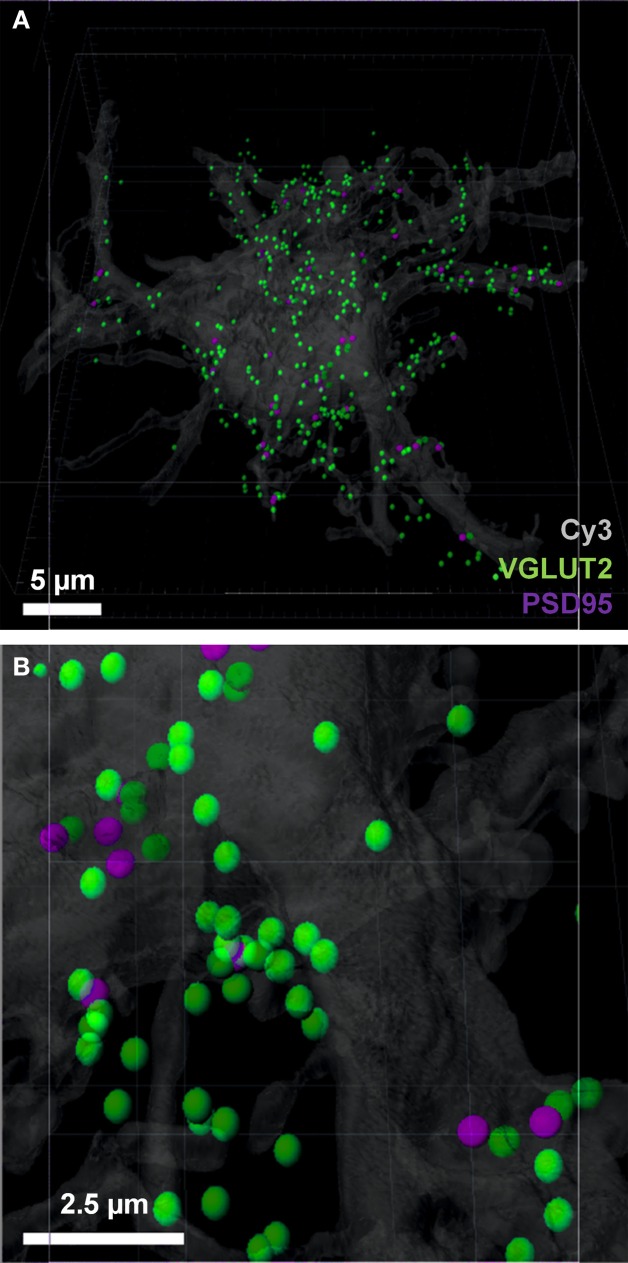
**Pre-synaptic and post-synaptic localizations to the filled motor neuron. (A,B)** show the pre-synaptic VGLUT2 (green) localized to within 1 μm of the neuronal surface and post-synaptic PSD-95 (purple) Imaris-generated “spots” with the neuronal surface. Evident is the increased amount of VGLUT2 puncta quantified using the neuron as the localization point, as opposed to the post-synaptic specialization used in Figure 3. Scale bars: **(A)** 5 μm and **(B)** 2.5 μm.

We chose the “*tools*” tab within the Imaris program, and selected the “*find spots close to surface*” option, to locate the total number of pre-synaptic VGLUT2 positive spots adjacent to the filled neuron (i.e., the first analytical option). This opened a Matlab dialog, which prompted the choice of spots to analyze; we chose the spot layer corresponding to the pre-synaptic channel. The distance threshold for analysis was chosen to be 1 μm, lying within the bounds (0.7–2.5 μm) established from multiple studies of synaptic morphology using similar material (Tamas et al., 2000; Issa et al., 2010; Ausdenmoore et al., 2011; Schatzle et al., 2012). Two new spot layers were then created by Imaris, one with all the pre-synaptic spots within 1 μm of the neuron surface, the other containing all the more distant spots; in this case, we detected 356 spots within 1 μm of the neuron (Figures 4A,B).

Using Imaris, we were also able to determine how many of these pre-synaptic spots were directly opposed to post-synaptic spots that were present within the cell surface of the filled neuron (i.e., the number of VGLUT2 positive synapses, the second analytical option). In order to do this, we selected the post-synaptic spot layer, chose the “*tools*” tab and selected the “*co-localize spots*” option. Once again, a Matlab dialog box was opened and we selected both pre- and post-synaptic spot layers in the menu. We chose a 1 μm maximum distance threshold between the spots for this analysis. Two new spot layers were created in Imaris containing the pre and post-synaptic spots found within 1 μm of each other, in this case we detected 45 pre-synaptic spots and 51 post-synaptic spots (Figures 3C,F).

### Quantification of glutamatergic and GABAergic synapses on hypoglossal motor neurons in P0 mice

**Figure 5:**
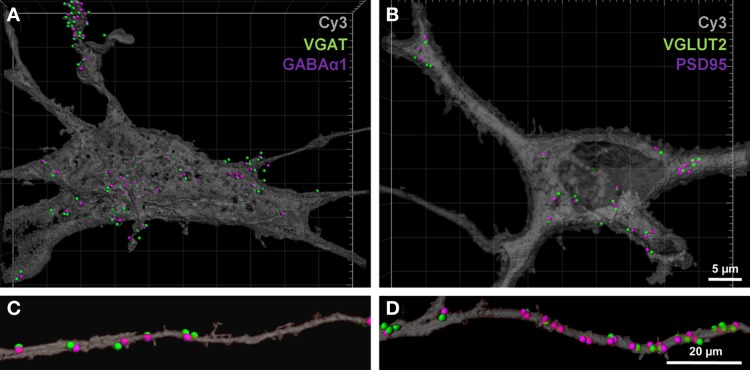
**GABAergic and glutamatergic synapses on the somas and distal dendrites of P0 hypoglossal motor neurons. (A)** shows a Neurobiotin-filled hypoglossal motor neuron soma and proximal dendrites double-labeled with inhibitory GABAergic synaptic components VGAT (green) and GABA-A receptor subunit α1 (purple). **(B)** shows a Neurobiotin-filled hypoglossal motor neuron soma and proximal dendrites double-labeled with excitatory glutamatergic synaptic components VGLUT2 (green) and PSD-95 (purple). Note that the GABAergic synapses are more prevalent than glutamatergic synapses on P0 hypoglossal motor neuron cell somas and proximal dendrites. **(C)** shows distal dendrite of hypoglossal motor neuron double-labeled with inhibitory GABAergic synaptic components VGAT (green) and GABA-A receptor subunit α1 (purple). **(D)** shows a distal dendrite of hypoglossal motor neuron double-labeled with excitatory glutamatergic synaptic components VGLUT2 (green) and PSD-95 (purple). Note that the glutamatergic synapses are more prevalent than GABAergic synapses on P0 hypoglossal motor neuron distal dendrites. Scale bar: **(A,B)** 5 μm and **(C,D)** 20 μm.

To determine the number of putative glutamatergic and GABAergic synapses onto hypoglossal motor neurons, we applied the above quantification method to several Neurobiotin-filled motor neurons. On motor neuron somas and proximal dendrites, the number of inhibitory VGAT pre-synaptic terminals within 1 μm of a post-synaptic GABA-A α1 receptor subunit was significantly greater than the number of excitatory glutamatergic VGLUT2 terminals (approximately five and a-half times more VGAT terminals than VGLUT2, *P* < 0.05; Figures 5A,B; Table 1). In the distal dendrites, the balance is reversed, with VGLUT2 terminals significantly more prevalent than VGAT terminals (approximately twice as many VGLUT2 terminals than VGAT, *P* < 0.05; Figures 5C,D; Table 1). On motor neuron somas and proximal dendrites, the number of inhibitory GABAergic post-synaptic GABA-A α1 receptor subunit spots was significantly greater than the number of excitatory glutamatergic post-synaptic PSD-95 spots (approximately four times more GABA-A α1 receptor subunit spots than PSD-95 spots, *P* < 0.05; Figures 5A,B; Table 1). The opposite was the case in the distal dendrites, where PSD-95 post-synaptic spots were significantly increased compared to GABA-A α1 receptor subunit spots (approximately twice the amount of PSD-95 as GABA-A α1 receptor subunit spots, *P* < 0.05, Figures 5C,D; Table 1).

**Table 1:** **Quantification of GABAergic and glutamatergic synapse components in P0 motor neuron somas and distal dendrites**.

**Location**	**VGAT spots (*n* = 6)**	**GABA-Aα1 spots (*n* = 6)**	**VGLUT2 spots (*n* = 4)**	**PSD95 spots (*n* = 4)**
Soma + proximal dendrites	212.1 ± 52.8	159.7 ± 38.2	39.3 ± 7.7	38.8 ± 9.1
Distal dendrites	18.8 ± 3.5	20.7 ± 4.3	41.8 ± 10.9	40.8 ± 7.7

Spots are quantified using the method described above, with VGAT and VGLUT2 pre-synaptic spots being within 1 μm of a GABA-A α1 receptor subunit or PSD-95 spot respectively. Data presented as mean ± SEM. Below, we quantify the number of spots of each type found in our sample at representative parts of the motor neuron; there is a higher proportion of inhibitory synapses found on the soma and proximal dendrites (PDs), while there is a higher proportion of excitatory synapses found on the distal dendrites.

### Specificity of immunocytochemistry

Next, we needed to validate that our method for co-localization of pre-and post-synaptic elements of excitatory glutamatergic and inhibitory GABAergic synapses was robust. We therefore tested the level of co-localization of the pre-and post-synaptic elements that defined either an excitatory or an inhibitory synapse. To do this, we performed two double immuno-labeling experiments on Neurobiotin-filled neurons. The first experiment was to locate excitatory and inhibitory pre-synaptic terminals in the one tissue slice, using anti-VGLUT2 and anti-VGAT antibodies, respectively. The second experiment was to locate excitatory and inhibitory post-synaptic specializations within the same tissue slice, using anti-PSD 95 and anti GABA-A α1 receptor antibodies, respectively.

**Figure 6:**
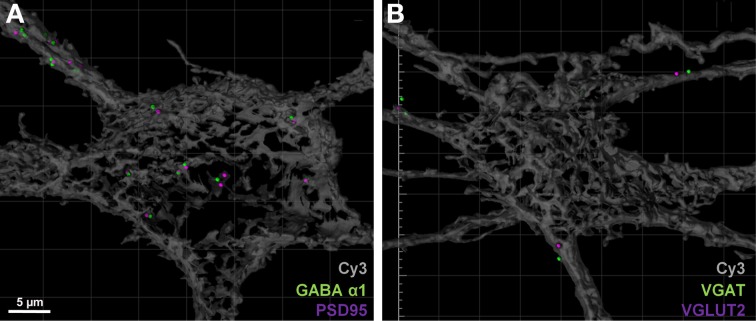
**Excitatory and inhibitory immuno-labels do not substantially overlap when localized to motor neurons. (A)** shows the co-localization (within 1 μm) of post-synaptic excitatory PSD-95 (purple) and inhibitory GABA-A receptor subunit α1 (green) on a Neurobiotin = filled hypoglossal motor neuron (gray). Only 15% of all PSD-95 spots are co-localized with a GABA-A receptor subunit α1 puncta. **(B)** shows the co-localization (within 1 μm) of pre-synaptic excitatory VGLUT2 (purple) and inhibitory VGAT (green) within 1 μm from the surface of a Neurobiotin = filled hypoglossal motor neuron (gray). Note that less than 10% of all VGLUT2 terminal boutons are co-localized with VGAT terminals. Scale bar: 5 μm.

For post-synaptic specializations of excitatory and inhibitory synapses, we observed approximately 15% of all PSD-95 puncta within the soma or dendritic arbor were co localized (threshold set at 1 μm) with GABA-A α1 receptor subunit puncta (Figure 6A, Table 2). Similarly, co-localization of excitatory and inhibitory VGLUT2 and VGAT pre-synaptic markers was approximately 10% when filtered to within a 1 μm radius of the filled motor neuron (Figure 6B, Table 2). By including these double antibody labeling experiments, we have provided supporting evidence that the double-labeling of sections with VGLUT2 and PSD-95 is a robust assay for quantifying excitatory glutamatergic synapses on Cy3-Neurobiotin filled hypoglossal motor neurons, with the ambiguity of potential excitatory or inhibitory synaptic site overlap being less than 15% using this method.

**Table 2:** **Specificity of immunocytochemistry**.

**Antibodies**	**Spots**	**Spots within 1 μm (co-label)**	**% co-label**
PSD-95 within cell *n* = 4	52.7 ± 13.9	7.5 ± 2.9 (GABA α1 co-label)	14.2
GABA-A α1 within cell *n* = 4	209.3 ± 55.4	12 ± 2.7 (PSD-95 co-label)	5.7
VGLUT2 within 1 μm of cell *n* = 3	466 ± 107.7	49 ± 26 (VGAT co-label)	10.5
VGAT within 1 μm of cell *n* = 3	473.4 ± 139	62.3 ± 26.1 (VGLUT2 co-label)	13.2

Table show spots of excitatory (PSD-95) and inhibitory (GABA-A α1) post-synaptic specializations within the same Neurobiotin-filled neuron somas and the number of spots of each synapse type within 1 μm of each other. Also shown are spots of excitatory (VGLUT2) and inhibitory (VGAT) pre-synaptic specializations within 1 μm of Neurobiotin-filled neuron somas and the number of spots of each synapse type within 1 μm of each other. Data presented as mean ± SEM.

### Super-resolution microscopy

**Figure 7:**
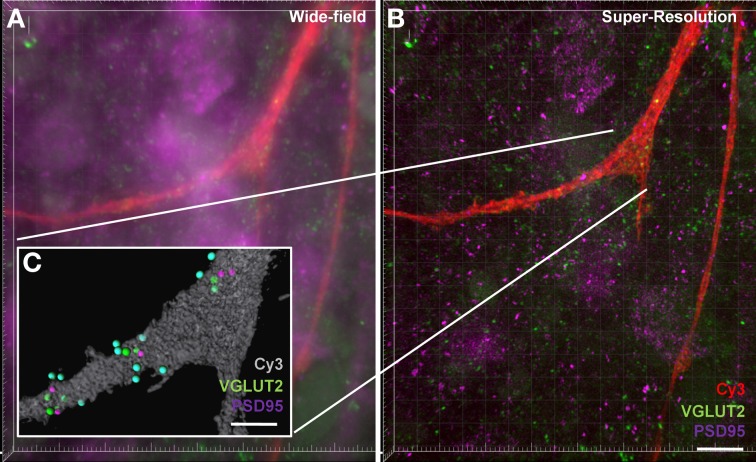
**Increased accuracy of synaptic component quantification by the super-resolution microscopy compared to the conventional confocal microscopy. (A)** shows a maximum intensity projection of a wide-field *z*-stack imaging Neurobiotin-filled hypoglossal motor neuron distal dendrites (red) with glutamatergic VGLUT2 (green) and PSD-95 (purple). **(B)** shows a maximum intensity projection of a super-resolution *z*-stack imaging of same distal dendrites (red) with glutamatergic synapse labeling with VGLUT2 (green) and PSD-95 (purple). Inset **(C)** shows the Imaris method when applied to the super-resolution sample, with the neuronal surface (gray), co-localized to within 1 μm of each other VGLUT2 (green) and PSD-95 (purple). VGLUT2 spots that are within 1 μm of the neuronal surface are colored blue. Scale bars: **(A,B)** 10 μm and **(C)** 5 μm.

Our final aim was to determine how our method could be applied to structured illumination microscopy (SIM). SIM is an emerging imaging super-resolution modality, offering twice the typical resolution of a conventional microscope (Gustafsson, 2000; Dani et al., 2010) and thus lending itself to synaptic analysis with improved spatial accuracy. Using SIM, we generated a three-color *z*-stack in a region containing a Neurobiotin-filled motor neuron and compared this to the analysis of the three-color z-stack for the same region generated using standard confocal microscopy (Figure 7). As expected, the improved resolution of SIM (~120 nm *xy*-plane) was able to resolve more discrete labeling of pre-and post-synaptic elements. Improved contrast aided in the delineation of various puncta providing a more thorough analysis of synaptic labeling (i.e., co localized pre-and post-synaptic immuno-labeling). This improvement in resolution translated to a more accurate co-localization distance for detecting pre- and post-synaptic interactions down to ~0.5 μm, thus reducing the chance for detecting false positive synapses. Our figure shows that even using a maximum intensity projection of a flat image (Figures 7A,B), staining that may have been observed as a single punctum (or spot) with conventional confocal imaging was “split” into two (or more) separate puncta by SIM imaging. In this sample data set, we also examined the co-localization of VGLUT2 and PSD-95. The super-resolved image can be seen compared to a wide-field image at the same site, along with the synaptic-component 3-dimensional projection (Figure 7). In this example, VGLUT2 spots within 1 μm are tinted blue to differentiate those co-localized to within 1 μm of a membrane-bound PSD-95 punctum (purple). Using SIM, the amount of glutamatergic synaptic puncta co-localized within 1 μm was increased compared to the same sample under regular confocal microscopy, (VGLUT2 spots: 14 using SIM, 13 using standard confocal; PSD-95: 11 using SIM, 8 using confocal).

### Electrophysiological recordings correlate with the synaptic labeling

**Figure 8:**
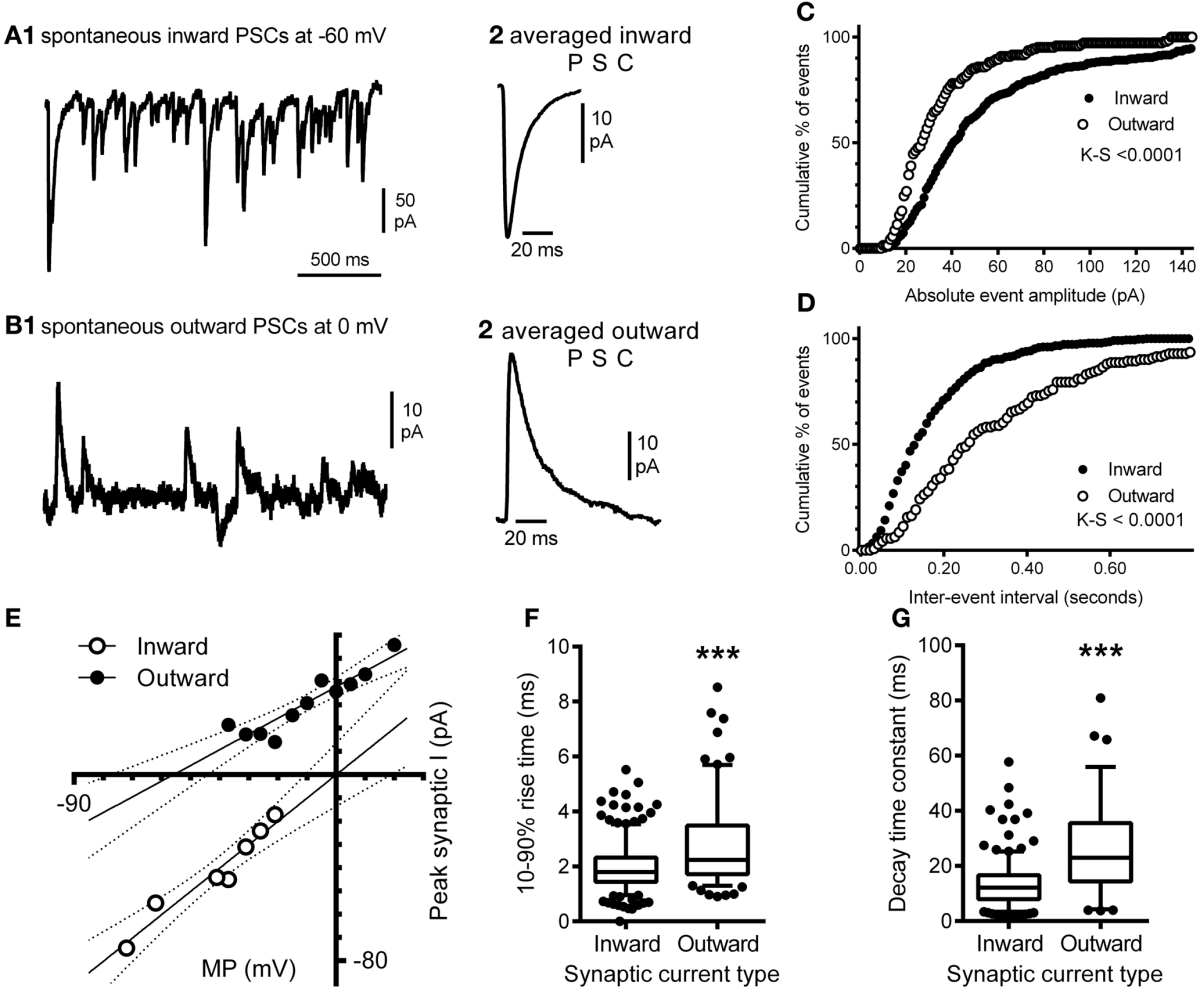
**Electrophysiological recording of inward and outward spontaneous synaptic currents shows correlation with excitatory and inhibitory synaptic labeling in hypoglossal motor neurons.** Inward synaptic currents are more frequent and have a different waveform to outward synaptic currents; **(A1)** shows a continuous recording of spontaneous inward synaptic currents at a membrane potential of −60 mV, while **(A2)** shows the averaged waveform of a single inward synaptic current, with a fast 10–90% rise time and decay time. **(B1)** shows a continuous recording of spontaneous outward synaptic currents at a membrane potential of 0 mV, from the same hypoglossal motor neuron shown in **(A)**, while **(B2)** shows the averaged waveform of a single outward synaptic current; note that the 10–90% rise time and decay time of the outward current are slower than seen in inward synaptic currents **(B2)**. Inward and outward synaptic currents arise from different populations of synaptic inputs to the hypoglossal motor neuron; **(C)** is the cumulative frequency distribution of the absolute amplitudes of all inward currents (*n* = 349) at −60 mV and outward currents (*n* = 142) at 0 mV recorded from the same neuron; the distributions are significantly different (Kolmogorov–Smirnov test, *P* < 0.0001), while **(D)** is the cumulative frequency distribution of the inter-event interval of all inward and outward currents from the same neuron; the distributions are also significantly different (Kolmogorov–Smirnov test, *P* < 0.0001). Inward synaptic currents have a positive reversal potential consistent with being excitatory glutamatergic currents, while outward currents have a negative reversal potential consistent with being inhibitory GABA_A_/glycinergic synaptic currents; **(E)** shows the current-voltage relationship of the mean amplitude of inward (open circles) and outward (filled circles) currents at different membrane potentials (MP); linear regression of current amplitude against MP (solid lines) shows that the slope and MP intersection for zero current are both significantly different; the dashed lines show the 95% confidence intervals for linear regression. Inward currents have a significantly faster 10–90% rise times and decay time constant, compared to outward currents; **(F)** shows that the distribution of inward and outward current 10–90% rise times from the same hypoglossal motor neuron are significantly different (*P* < 0.0001), as are the distributions of decay time constants for the same currents **(G)**. Both data sets **(F,G)** are shown as box-and-whiskers plots, where the box shows the mean, whiskers are from the 5 to 95 percentile, and circles show outliers below or above these limits. ^***^P <0.0001, Student *t*-test.

Our morphological study found that the balance between excitatory and inhibitory synapses onto the hypoglossal motor neurons of a P0 mouse favored excitation, as the dendritic arbor, with significantly greater excitatory inputs than inhibitory, accounts for over 97% of the volume of the motor neuron (Ulfhake and Kellerth, 1981). Here, we show evidence that this is commensurate with functional electrophysiological data. In a recorded P0 mouse hypoglossal motor neuron at a holding potential of −60 mV, spontaneous synaptic currents were all inward (Figures 8A1,A2) and were frequent (mean frequency of 9.8 ± 0.5 Hz), with a mean 10–90% rise time of 1.96 ± 0.04 ms (Figure 8F) and decay time constant of 13.1 ± 0.5 ms (Figure 8G). By contrast, at a holding potential of 0 mV, spontaneous synaptic currents were predominantly outward (Figures 8B1,B2) and were less frequent (mean frequency of 5.3 ± 0.5 Hz; *P* < 0.0001), with significantly longer 10–90% rise time (Figure 8F; mean of 2.72 ± 0.12 ms; *P* < 0.0001) and decay time constant (Figure 8G; mean of 26.1 ± 1.8 ms; *P* < 0.0001). The cumulative frequency distributions for the absolute amplitude (Figure 8C) and inter-event interval (Figure 8D) of inward and outward currents at −60 mV and 0 mV, respectively were also significantly different (Kolmogorov–Smirnov test, *P* < 0.0001 for both). The reversal potentials for inward and outward synaptic currents were determined by holding the hypoglossal motor neuron at different potentials, recording an epoch of data, and measuring the average amplitude of outward and inward synaptic currents (Figure 8E). Linear regression of holding potential against current amplitude (dashed lines in Figure 8E) found that the reversal potential for inward currents was 0 mV (95% confidence interval from −13 to +13 mV), while the reversal potential for outward currents was −56 mV (95% confidence interval from −77 to −44 mV). The measured inward current reversal potential was not significantly different from the calculated reversal potential for a glutamatergic EPSC, while the measured outward current reversal potential was not significantly different from the calculated reversal potential for a Cl^−^-dependent IPSC.

### Imaging and methodological considerations

**Figure 9:**
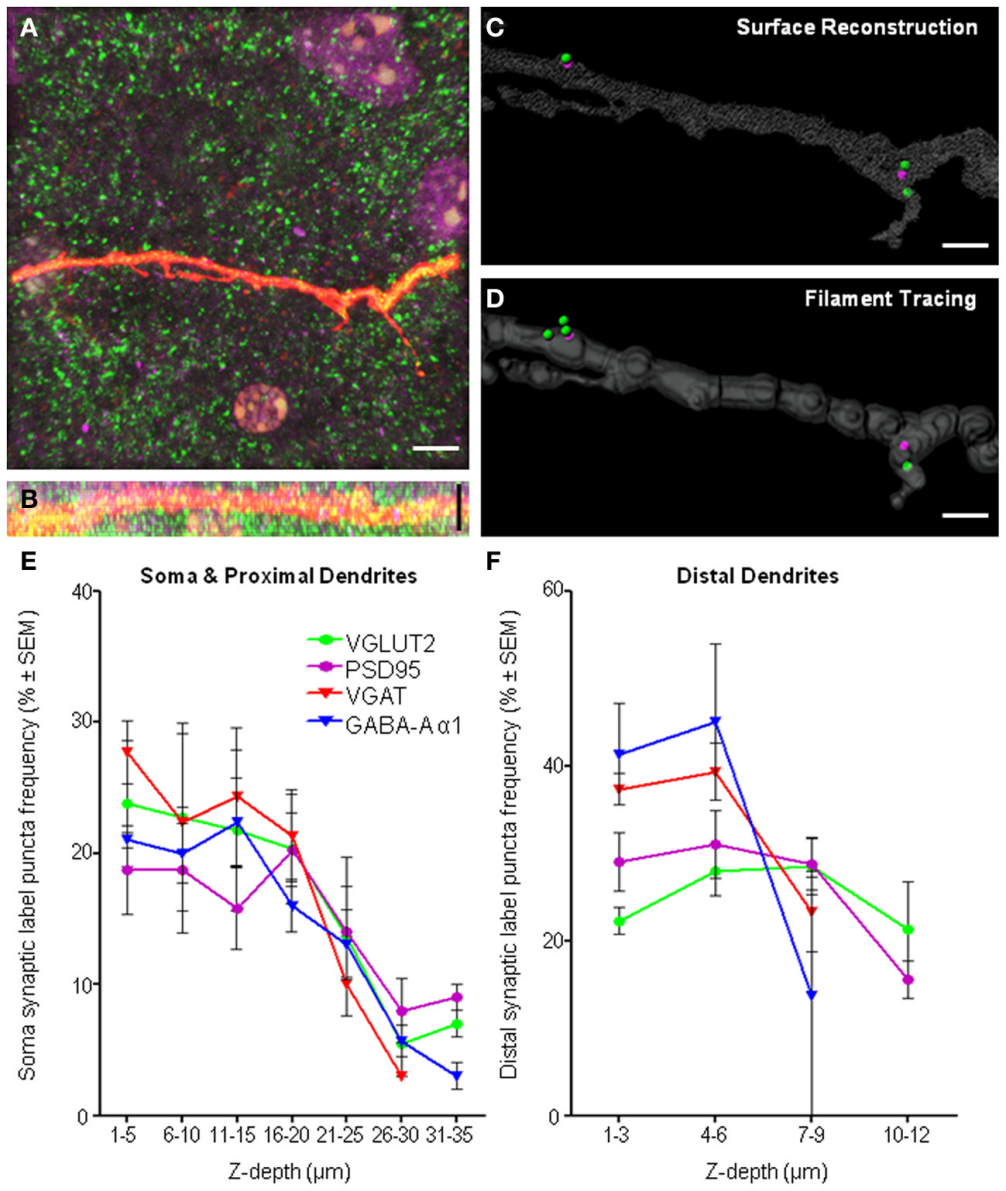
**Imaging and methodological considerations. (A)** Shows a maximum intensity projection in the *xy*-axis of a representative distal dendrite (red fluorescence) and excitatory pre-synaptic VGLUT2 (green) and post-synaptic PSD-95 (purple). **(B)** Shows the same fluorescence in the *xz*-axis, indicating penetration of all immuno-labeling within dendritic *z*-stacks. We show the same dendrite with our above method applied to quantify synaptic components using two different methods of defining the dendritic volume, namely, surface reconstruction **(C)** and filament tracing **(D)**. We show that equivalent results with regard to synapse quantifications using either surface reconstruction filtering or filament trace filtering. For surface filtering of data, 2 spots internal (PSD-95), 3 spots external (VGLUT2), 2 synapses in total. For filament trace filtering of data, 2 spots internal (PSD-95), 4 spots external (VGLUT2), 2 synapses in total. Frequency histogram analysis of “spot detection” labeling density (%) for all immuno-labels and all regions, the soma and proximal dendrites **(E)** and distal dendrites **(F)** illustrated the effective depth of antibody penetration from the top of the confocal *z*-stack. Note the top of the confocal stack was some depth below, up to 20 μm from the surface of tissue slice. For each immuno-label (i.e., colored line), the number of puncta in each depth bin (binned in 5 μm increments for the soma and 3 μm increments in the distal dendrites) was calculated as a percentage of the total number of puncta at all depths sampled (reading *x*-axis from left to right). In regard to the distal dendrites **(F)**, the smaller *z*-depth shown was not due to poor antibody penetration, but rather, was due to the smaller *z*-stack required to image distal dendrites, as our sampling ended ~2 μm below the dendrite of interest [see **(B)** as a typical example of the shallow *z*-depth of a distal dendrite. Thus, the puncta frequency distribution for distal dendrites was largely within the first 6 μm of *z*-depth. Scale bars: **(A,B)** 10 μm and (**C,D)** 2 μm.

As our study involved the imaging of fluorophores at varying depths within a tissue slice, there is a clear need to determine whether the general gross morphology of the neuron of interest could be adequately resolved, with particular attention to the *z*-axis distribution of the dendritic arbor of the Neurobiotin-filled cell within the slice. The resolution of distal dendrites and VGLUT2 and PSD-95 immuno-labels in the *xy*-plane (Figure 9A) and the *xz*-plane (Figure 9B), showing good antibody penetration within the first 20–30 μm from the top of the collected confocal *z*-series. In our slice preparations the top of the confocal stack was up to 20 μm below the surface of the tissue. This distance is relative to the labeled cell, and the top of the neuron and dendrites may be anything up to 20 μm from the surface of the tissue slice, thus necessitating the need to control for depth-related experimental limitations. Despite our best efforts to control for these limitations, some practical and methodological deficiencies remain inherent in our study, namely limitations with the *z*-resolution, which can lead to neuronal somatic and dendritic reconstruction distortion along the *z*-axis, and penetration of the antibody labels deeper into the tissue slice.

Creation of a surface directly over a fluorescent signal is known to cause an anisotropy and overestimation of surface area and volume (Kubinova and Janacek, 2001). This is a result of fundamental aberration in the point-spread function, elongated in the *z*-axis of confocal microscopy (Shaw, 1994), causing anisotropy of the reconstructed neuron and potential overestimation of post-synaptic labels and therefore synapse number. Researchers must be aware of this issue in their analysis, and understand it may cause an overestimation of the number of structures localized within a dendritic proximity. Errors caused by this reduced *z*-resolution in confocal imaging may be addressed by tracing dendrites using superior algorithms accounting for the anisotropy of the dendritic projections by using generalized cylinders with circular cross sections (Schmitt et al., 2004). We used both types of neuronal tracing on distal dendrites in a subset (*n* = 4) of our sampled motor neurons, and found little difference comparing our above surface method (Figure 9C) with the general cylinder approximation filament tracing algorithm from Imaris software (Figure 9D) with dendritic diameter defined by local contrast and with a minimal diameter of 0.4 μm (dendritic volume: 144.25 ± 25.41 μm^3^ surface method, 135.75 ± 33.41 μm^3^ filament method, *P* = 0.41. Presynaptic VGLUT2: 8.25 ± 3.04 spots surface method, 7.75 ± 2.25 spots filament method, *P* = 0.89. Postsynaptic PSD-95: 4.75 ± 1.55 spots surface method, 4.75 ± 1.38 spots filament method, *P* = 1. All analyses were paired *t*-tests).

Applying our methodology to neurons, which necessitates imaging deeper into tissue sections would require a long working distance objective, such as a glycerol immersion objective. However, in our case, other factors had already limited our analysis to structures within the first 50 μm (or in some cases less) from the coverslip. These limiting factors, apart from the working distance of the oil objective, are light scattering, tissue transparency, and antibody penetration. Readers should ensure they address each of these factors when designing their study, and be aware of the limitations in their images. Frequency histogram analysis of labeling density as a function of structure depth in the *z* axis is a useful way to screen data for any inconsistencies or systematic biases related to tissue depth. In our case, we did this for all immuno-labels (i.e., colored lines) and for all regions, including the soma and proximal dendrites (Figure 9E) and distal dendrites (Figure 9F). These frequency histograms were generated by counting the number of puncta at each depth bin (soma and proximal dendrites binned using 5 μm increments and distal dendrites were binned at 3 μm increments) and calculating this as a % of the total number of puncta at all depths sampled (reading the *x*-axis from left to right in Figures 9E,F). In our case, the top of the optical *z*-stack, which could be up to 20 μm below the tissue surface, has been designated 0 μm, so all measurements were relative to the imaging process, and not to the surface of the tissue slice. Data sets generated for each pre- and post-synaptic pair were examined and the immuno-label with the steepest decline (and therefore the worst penetration) was used to determine the maximum *z*-depth with antibody penetration suitable for analysis. This depth threshold was set by excluding all analysis beyond the cumulative frequency of 85% (i.e., before the decline in “spot” detection was too steep). In the case of the soma, occasionally less than the entire volume was suitable for analysis, whereas for distal dendrites, all dendritic volumes were within the region deemed suitable for analysis.

### Comparison to electron microscopy

Morphometric analysis of synapse numbers using electron microscopy techniques is the “gold standard” for synapse quantification in neurons (Mishchenko et al., 2010; Dumitriu et al., 2012). Pioneering work has linked pre-synaptic structures (vesicular formation and active zone) and post-synaptic densities at the ultra-structural level to defined sites on the neuron soma-dendritic axis (Gray, 1959; Uchizono, 1965; Colonnier, 1968), see (Klemann and Roubos, 2011) for a recent review on this topic). The terms asymmetric synapses (also known as Type I and nominally excitatory) and symmetric synapses (Type II and nominally inhibitory) derive from the same studies. Estimates of the density and number of total synapses and two subdivisions of asymmetric and symmetric synapses in the rat hypoglossal nucleus, taken from electron microscopy of random sections(O'Kusky, 1998) provides a basis for comparison to our results. As electron microscopy synapses are all in a 1:1 pres-synaptic/post-synaptic ratio, and our data was reported related to spot detection of individual pre and post-synaptic components, we converted our data to density measurements to compare our results with previous electron microscopic synaptic densities. The correlation between electron microscopy synapse quantification studies with those using confocal microscopy has been validated previously (Hohensee et al., 2008). The first comparison was the total synaptic density of our filled neurons, compared to the total synaptic density of the rat hypoglossal nucleus. The mean synaptic density of glutamatergic plus GABAergic synapses on the filled hypoglossal motor neuron was 129.9 million synapses per mm^3^, 75.1% of the number reported for rat P1 hypoglossal motor nucleus as a whole [147 million synapses per mm^3^; (O'Kusky, 1998)]. We then compared our excitatory glutamatergic densities on filled hypoglossal motor neuron distal dendrites with asymmetric synapses found on the axospinous and axodendritic compartments [corresponding to dendritic shafts and spines, respectively; (O'Kusky, 1998)] of the rat hypoglossal motor nucleus and our method matched within 2% the of electron microscopy estimate at P1 (O'Kusky, 1998). When comparing GABAergic synapses on our filled hypoglossal motor neuron with symmetric synapse densities obtained by the previous electron microscopy study, our estimate was 14% greater than that obtained by electron microscopy.

We also did an estimation of synapse density based on the entire volume of our confocal *z*-stack, analysing all of the synapses within the stack. Based on our above “spot-detection” criteria without filtering for the filled neuron gave an overall excitatory and inhibitory synapse density estimate. For each pair of excitatory and inhibitory synaptic markers, we calculated the synapse density in mm^3^. Comparing back to the synaptic density measured by electron microscopy in rat P1 hypoglossal nucleus, our total synapse density was 92% of the electron microscopy density; our glutamatergic synapse estimate was 106% of the asymmetric synapse density obtained using electron microscopy; and our GABAergic synapse estimate was 82% of that found using electron microscopy [data derived from O'Kusky (1998)]. In the case of symmetric synapses, the underestimation is likely to be due to glycinergic inhibitory inputs, known to occur on hypoglossal motor neurons (Berger, 2000), which we would not detect as we used a GABAergic post-synaptic marker.

Although the rat is a different species, we believe our results are in reasonable accord with these previous findings, allowing for our under-estimate of inhibitory synapses compared to electron microscopy and our methodological limitations, with particular regard to precise surface delineation of somas and dendrites which potentially overestimates all synapse numbers, regardless of type.

## Discussion

The present study provides a description and validation of Imaris 3D reconstructions of glutamatergic (excitatory) and GABAergic (inhibitory) synaptic inputs to individual motor neurons in an intact brainstem slice. We also present results matching these morphologic synaptic quantifications with functional assessment of excitatory and inhibitory synapses onto motor neurons. Our method provides a standardized way of quantifying various synaptic components on motor neurons after electrophysiological recording. This method preserves the majority of inputs onto both the motor neuron soma and its dendritic processes. We believe it is an improvement on previous methods used to locate and count the number of synapses on motor neurons *in vivo*, as it provides for a relatively rapid quantification of synapses and their neuro-chemical type in combination with electrophysiological analyses of synaptic activity.

Previous studies aimed at analyzing the number and type of synapses made onto motor neurons, include: retrograde labeling of motor neurons with subsequent immuno-staining (e.g., Mantilla et al., 2009); impalement of motor neurons with sharp electrodes, filling with Neurobiotin, cryo-sectioned at 50 μm, followed by immuno-staining for pre-synaptic endings (e.g., VGLUT1), then analyses for the location of immuno-labeled pre-synaptic elements on the surface of motor neurons using a center-distance algorithm (Ausdenmoore et al., 2011); and the time consuming technique of electron microscopy to locate various neurotransmitters within pre-synaptic endings and their location with respect to the surface of motor neurons (O'Kusky, 1998; Shigenaga et al., 2005).

While all these studies have been informative, they all lack the ability to define the synapse at the molecular level, namely by the apposition of specific pre-and post-synaptic elements with respect to the surface of a labeled neuron. Our study addresses this major limitation by employing imaging software techniques that has allowed us to locate immuno-labeled pre- and post-synaptic elements within the one section, thereby defining synaptic type, and their distribution over the surface of an individually filled motor neuron, namely its soma and dendrites. All this is achieved in a single tissue slice that has captured the neuron's soma and the majority of its dendritic field, avoiding reconstructions from post-processed serially cut sections, which in turn have to be stained, reconstructed, and analyzed. In addition, by employing strict thresholding and distance criteria that are a feature of most image analysis programs, our approach can also largely eliminate observer bias. Our results suggest that quantifying synapses with regard to only pre-synaptic neurotransmitter release boutons is prone to an overestimate of synapse number, as there was a far greater number of VGLUT2 pre-synaptic labels localized to within 1 μm of cell (307.5 ± 62.8) than that co-localized to within 1 μm of a post-synaptic PSD-95 spot (39.3 ± 7.7). While it has been shown using electron microscopy techniques that a small proportion (4%) of hippocampal terminal boutons lack a post-synaptic specialization (Harris and Weinberg, 2012), the magnitude of the difference using the two quantification methods supports the use of co-labeling pre- and post-synaptic components to more accurately estimate synapse numbers. Furthermore, the gross estimation of synapse numbers based upon pre-synaptic neurotransmitter labeling may hide the subtleties present in synaptic formations, as the ratio of pre-synaptic label to post-synaptic label is not quite 1:1, perhaps indicating that multiple release sites may share post-synaptic domains [multi-synaptic boutons (Harris and Weinberg, 2012)], and/or post-synaptic specializations may be trafficked to the somatic or dendritic membrane without contact with a pre-synaptic release site (Rao and Levi, 2000).

Increased optical resolution of fluorescent microscopy by using super-resolution techniques such as SIM indicate that enhanced resolution increases the amount of pre- and post-synaptic puncta detected using our criteria (Section Super-Resolution Microscopy). This is possibly due to the increased ability to discern two different but closely juxtaposed puncta. Although this indicates that using conventional confocal imaging and our method somewhat underestimates the number of synapses, the overall magnitude of the difference in synapse number using conventional and super-resolution techniques (8% increase in VGLUT 2 co-localized to PSD-95 distal dendritic spots with SIM super-resolution method compared to regular confocal) is of a much lesser magnitude than that of assaying pre-synaptic neurotransmitter alone (23% increase in VGLUT2 spots when quantifying to within 1 μm of distal dendrites compared to quantifying within 1 μm of a PSD-95 spot).

### Limitations

Our method has some inherent assumptions and limitations beyond those discussed and accounted for in the results; these include the nature of the putative synapse, the small yet measurable co-localizations between some inhibitory and excitatory markers (section Specificity of Immunocytochemistry), the dimensions of the immuno-labeled puncta, the uniform maximal distance between the pre- and post-synaptic domains of different synapse types and antibody penetration and background fluorescence issues.

The labels we have chosen to use were meant to provide a robust, repeatable immune-label for putative excitatory and inhibitory synapses, but are by no means exhaustive in regard to either GABAergic or glutamatergic synapses. In principle, our method could use other pre- and post-synaptic markers for quantification of synapse number, such as GAD65 and GAD67 for GABAergic (Szabadits et al., 2011; Cserep et al., 2012) and VGLUT1 for glutamatergic pre-synaptic markers (Bae et al., 1999; Herzog et al., 2004; Shigenaga et al., 2005; Issa et al., 2010). Other potential pre-synaptic labels include; piccolo, a protein that anchors synaptic vesicles to synaptic terminals (Cases-Langhoff et al., 1996), bassoon, concentrated in active zones and co-localized with synaptic vesicles and piccolo (Tom Dieck et al., 1998), VAChT (vesicular acetylcholine transporter), which transports acetylcholine to secretory vesicles (Weihe et al., 1996), serotonin, a widespread neurotransmitter with excitatory effects upon hypoglossal motor neurons (Aldes et al., 1989; Berger et al., 1992). Other post-synaptic labels that may prove useful includes; glycine receptor, the inhibitory amino acid neurotransmitter receptor (Lynch, 2004), gephyrin, responsible for membrane clustering of glycine and some GABA receptors (Kirsch and Betz, 1995), NMDA (Moriyoshi et al., 1991) and AMPA (Prithviraj et al., 2008) glutamate receptors, and Pick, which interacts with AMPA receptors and PSD-95 (Xia et al., 1999).

A strict separation between glutamatergic and GABAergic inputs is commonly thought to occur (Edwards, 2007). Our study revealed some degree of co-localization between pre- or post-synaptic excitatory and inhibitory markers used in our validation experiments (section Specificity of Immunocytochemistry). Although the degree of co-localization was relatively low (10–15%), we believe this is not due to imaging and methodological limitation, but reveals an important intrinsic characteristic of neurons. One possibility is that this represents true co-localization at the same synapse. There is emerging evidence suggesting that a subset of excitatory neurons show co-expression of VGAT and VGLUT2 in single synaptic terminals (Ottem et al., 2004; Zander et al., 2010). Co-release of glutamate and GABA from single nerve terminals has been demonstrated (Boulland et al., 2009). There is also evidence that excitatory postsynaptic densities may also contain a lower density of GABA A receptors, which can respond to co-released GABA (Bergersen et al., 2003). In addition, it is possible that co-localization could be due to either close anatomical apposition of distinct excitatory and inhibitory synapses on motor neurons, or to the presence of axo-axonic type synapses in which GABAergic synapses make contact with, and regulate pre-synaptic release from, glutamatergic synapses (Ruiz et al., 2003, 2010; Safiulina et al., 2006).

The dimensions of the puncta for the different immuno-labels are based on past immuno-labeling studies (Nahmani and Erisir, 2005; Waataja et al., 2008). The distance of 1 μm as the threshold for the co-localization of pre-synaptic markers with a post-synaptic label within the soma or dendrite of the motor neuron is supported by the multiple studies from similar material, with thresholds set from between 0.7 to 2.5 μm (Tamas et al., 2000; Issa et al., 2010; Ausdenmoore et al., 2011; Schatzle et al., 2012). Provided the assumed dimensions are applied consistently across all experimental conditions studied, the estimate of synaptic number should be reliable with regard to co-localization of pre and post-synaptic components, providing estimates of changes based on experimental manipulation. Of course, a pre-synaptic label being within 1 μm of a post-synaptic label on the Neurobiotin-filled cell soma or dendritic arbor is inconclusive evidence of a functional synapse, and may instead be in close proximity to the filled cell, but forming a synapse with another nearby cell.

### Comparison of synaptic quantification, electrophysiological assessment, and electron microscopy results

Here we summarize the quantification of excitatory and inhibitory synaptic inputs onto single hypoglossal motor neurons using our method, the electrophysiological assessment of the synaptic currents within the same neurons and compare these results to a previous hypoglossal motor neuron electron microscopy study. From our results, we show that the ratio between excitatory synapses and inhibitory synapses in hypoglossal motor neurons is roughly 2.17:1 in the distal dendrites (Table 1). Although inhibitory GABAergic synapses are far more prevalent than excitatory glutamatergic synapses at the soma, over 97% of the entire motor neuron is dendritic (Ulfhake and Kellerth, 1981, 99% this study), thus the synaptic inputs in the dendritic compartment make up the vast bulk of all synapses onto the motor neuron (favoring glutamatergic over GABAergic in a ratio of 2.17:1).

Our electrophysiological recordings show that EPSCs are more frequent than IPSCs (Section Electrophysiological Recordings Correlate with the Synaptic Labeling, Figures 8A1,A2,B1,B2). The ratio of this frequency difference of EPSCs to IPSCs is 1.85:1. This is commensurate with the overall ratio of labeled excitatory glutamatergic synapses compared to inhibitory GABAergic synapses. The most likely explanation for the slight difference in ratios derived from immune-labeling of synapses vs. functional recordings is the presence of inhibitory glycinergic IPSCs, which increase the number of outward currents and thus decrease the excitatory: inhibitory ratio. While our recordings were made from the motor neuron soma, and EPSPs from distal dendrites suffer passive attenuation as they travel to the soma, the correspondence in anatomical and functional estimates of excitatory: inhibitory input ratio suggests that EPSCs originating in distal dendrites do make a contribution to the synaptic current at the soma. Indeed, recent studies suggest several mechanisms which counter-act dendritic EPSC attenuation. EPSCs in small distal dendrites may be much larger in size than somatic EPSCs (Williams and Stuart, 2002), while dendritic membrane currents may be active modulators of synaptic input amplitude and time course (see Magee, 2000; Reyes, 2001; Spruston, 2008 for current reviews). In particular, EPSC propagation from distal dendrites to the soma can be enhanced by the presence of persistent Na^+^ current (Magee and Johnston, 1995) or the hyper-polarization activated cationic current I_H_ (Williams and Stuart, 2000), both of which are present in hypoglossal motor neurons (Binder, 2002; Heckman et al., 2003, 2004, 2008; Van Zundert et al., 2008; Ireland et al., 2012). We thus interpret our electrophysiological recordings, with a ratio of EPSCs to IPSCs of 1.85:1 to be commensurate with increased distal glutamatergic synapses in dendrites.

In the P1 rat hypoglossal nucleus, the ratio of asymmetric synapses (nominally excitatory) to symmetric synapses (nominally inhibitory) was reported to be 1.66:1 (O'Kusky, 1998). Similarly to our electrophysiological data, the lower ratio of excitatory to inhibitory synapses in the electron microscopy study when compared to our immuno-labeling data is most likely due to the contribution of glycinergic and other synapse types.

Overall, there is a very close correlation between our immuno-labeled estimates of glutamatergic vs. GABAergic synapses, our electrophysiological estimates of EPSC and IPSCs and previous electron microscopy studies of asymmetric and symmetric synapses, and these data combined suggest that the balance of excitation/inhibition on hypoglossal motor neurons favors excitation.

### Glutamatergic and GABAergic synapses can be morphologically and electrophysiologically assessed in single neurons

Our data quantifies the pre- and post-synaptic components of both GABAergic and glutamatergic inputs on motor neuron somas and distal dendrites, and relates this to electrophysiological synaptic currents. The ability of neuroscientists to reconcile synaptic function with morphology, with particular consideration to the dendritic contributions to synaptic signaling is therefore a distinct advantage in investigating functional perturbations and/or aberrant neurotransmitter signaling in many neurobiological diseases including autism, schizophrenia and epilepsy (Esclapez and Houser, 1999; Ramirez and Gutierrez, 2001; Wong et al., 2003; Ramocki and Zoghbi, 2008).

### Future directions

It is important to note that our method does not supersede synaptic quantification by electron microscopy, which still remains the gold standard for defining presence of a synapse. With the advent of new imaging technologies and tissue clearing methodologies, our analysis technique is likely to become increasing applicable to high throughput neuronal analysis. Future researchers should take advantage of newly developed tissue clearing methods creating transparent cell membranes (Erturk et al., 2012; Chung et al., 2013). These methods combined with imaging techniques capable of rapidly acquiring large fields of view in deep sections (e.g., glycerol objectives, spinning disc confocal microscopy and super resolution techniques, such as SIM as shown in this paper) will provide increased accuracy in delineating neuronal structure and identifying receptor sites. This will potentially allow our method to be applied to neurons located deep within tissue blocks or the intact brain. However, we caution readers to be aware of potential image artifacts that may arise using these techniques, and to routinely include control measures, such as those used here and elsewhere (Evers et al., 2005).

Even without using the aforementioned technological advances, enhancements of this method could include; (i) the *post-hoc* re-sectioning of the Neurobiotin-filled tissue slice into 50 μm sections that will allow for deeper neuronal soma and dendrites be assessed for synaptic components. (ii) Use of more sophisticated neuronal tracing algorithms, as outlined previously (Schmitt et al., 2004; Evers et al., 2005), which may produce a more accurate synaptic quantification.

We believe that our method provides a rapid assessment of synapse numbers on neuronal somas, proximal dendrites and distal dendrites, and that reliable synapse quantification in mice (comparing control groups with experimental models) is vital, as transgenic strains of species are a well-established pre-clinical model for a variety of neurologic disorders (Gondo, 2013) and the wild type mouse is frequently the default choice when looking for mammalian mechanisms of basic neuronal phenomena (Anagnostopoulos et al., 2001).

## Conclusion

In this study, we present a robust, repeatable, and relatively automated method for locating and quantifying the pre- and post-synaptic components on neuronal somata and dendrites. We provide data showing that this method is sensitive enough to differentiate excitatory and inhibitory components, to a tolerance of 15%. This method is likely to be useful in studies that combine electrophysiological and morphological analysis of single neurons, compatible with the increased resolution power of emerging imaging modalities such as found in super-resolution microscopy.

### Conflict of interest statement

The authors declare that the research was conducted in the absence of any commercial or financial relationships that could be construed as a potential conflict of interest.
